# 1,10-Phenanthroline-5,6-Dione–Based Compounds Are Effective in Disturbing Crucial Physiological Events of *Phialophora verrucosa*

**DOI:** 10.3389/fmicb.2017.00076

**Published:** 2017-01-30

**Authors:** Marcela Queiroz Granato, Diego de Souza Gonçalves, Sergio Henrique Seabra, Malachy McCann, Michael Devereux, André Luis Souza dos Santos, Lucimar Ferreira Kneipp

**Affiliations:** ^1^Laboratório de Taxonomia, Bioquímica e Bioprospecção de Fungos, Instituto Oswaldo Cruz, Fundação Oswaldo CruzRio de Janeiro, Brazil; ^2^Laboratório de Investigação de Peptidases, Departamento de Microbiologia Geral, Instituto de Microbiologia Paulo de Góes, Universidade Federal do Rio de JaneiroRio de Janeiro, Brazil; ^3^Programa de Pós-Graduação em Bioquímica, Instituto de Química, Universidade Federal do Rio de JaneiroRio de Janeiro, Brazil; ^4^Laboratório de Tecnologia em Cultura de Células, Centro Universitário Estadual da Zona Oeste (UEZO)Duque de Caxias, Brazil; ^5^Chemistry Department, Maynooth University, National University of IrelandMaynooth, Ireland; ^6^Centre for Biomimetic and Therapeutic Research, Focas Research Institute, Dublin Institute of TechnologyDublin, Ireland

**Keywords:** *Phialophora verrucosa*, 1,10-phenanthroline-5,6-dione, metal-based drugs, antifungal activity, chromoblastomycosis

## Abstract

*Phialophora verrucosa* is a dematiaceous fungus able to cause chromoblastomycosis, phaeohyphomycosis and mycetoma. All these fungal diseases are extremely difficult to treat and often refractory to the current therapeutic approaches. Therefore, there is an urgent necessity to develop new antifungal agents to combat these mycoses. In this context, the aim of the present work was to investigate the effect of 1,10-phenanthroline-5,6-dione (phendione) and its metal-based derivatives [Ag(phendione)_2_]ClO_4_ = ([Ag(phendione)_2_]^+^) and [Cu(phendione)_3_](ClO_4_)_2_.4H_2_O = ([Cu(phendione)_3_]^2+^) on crucial physiological events of *P. verrucosa* conidial cells. Using the CLSI protocol, we have shown that phendione, [Ag(phendione)_2_]^+^ and [Cu(phendione)_3_]^2+^ were able to inhibit fungal proliferation, presenting MIC/IC_50_ values of 12.0/7.0, 4.0/2.4, and 5.0/1.8 μM, respectively. [Cu(phendione)_3_]^2+^ had fungicidal action and when combined with amphotericin B, both at sub-MIC (½ × MIC) concentrations, significantly reduced (~40%) the fungal growth. Cell morphology changes inflicted by phendione and its metal-based derivatives was corroborated by scanning electron microscopy, which revealed irreversible ultrastructural changes like surface invaginations, cell disruption and shrinkages. Furthermore, [Cu(phendione)_3_]^2+^ and [Ag(phendione)_2_]^+^ were able to inhibit metallopeptidase activity secreted by *P. verrucosa* conidia by approximately 85 and 40%, respectively. Ergosterol content was reduced (~50%) after the treatment of *P. verrucosa* conidial cells with both phendione and [Ag(phendione)_2_]^+^. To different degrees, all of the test compounds were able to disturb the *P. verrucosa* conidia-into-mycelia transformation. Phendione and its Ag^+^ and Cu^2+^ complexes may represent a promising new group of antimicrobial agents effective at inhibiting *P. verrucosa* growth and morphogenesis.

## Introduction

*Phialophora verrucosa* is a melanized pathogenic fungus associated with a wide range of neglected diseases including phaeohyphomycosis, mycetoma, keratitis, endophthalmitis, osteomyelitis and endocarditis (Turiansky et al., 1995; Revankar and Sutton, 2010; Sun et al., 2010; Tong et al., 2013). However, this fungus is especially known to cause chromoblastomycosis (CBM), which is a chronic, progressive disease affecting the cutaneous and subcutaneous tissues (Torres-Guerrero et al., 2012; Krzyściak et al., 2014). Clinically, CBM is characterized by pseudoepitheliomatous hyperplasia with epidermal microabscesses and dermal granuloma. Chronic CBM lesions may undergo neoplastic transformation leading to skin cancer (Queiroz-Telles and Santos, 2012). This disease is most prevalent among individuals with outdoor occupations, such as farmers, gardeners and agricultural laborers. This risk group is usually exposed to soil, wood and rotting vegetation, which are the ubiquitous natural habitats of the fungal etiologic agents of CBM (Torres-Guerrero et al., 2012; Vicente et al., 2014). CBM usually occurs through trauma or skin penetration of fungal propagules in individuals lacking adequate protective footwear and clothing (Torres-Guerrero et al., 2012). This widespread mycosis is mostly common in tropical and subtropical regions of Africa, Asia, Australia and Latin America, with particular foci in Brazil, Madagascar, Mexico, Dominican Republic, Venezuela and India (Ameen, 2010; Krzyściak et al., 2014). Due to the chronic nature and the well-known multidrug-resistance profile, it is very difficult to treat patients with CBM using currently available therapies (Queiroz-Telles and Santos, 2012). Prolonged treatment and disease relapsed are huge concerns, and antifungal therapies especially for *Phialophora* infections have generally been disappointing (Gao et al., 2013). For these reasons, new antifungal agents should be studied in order to find out alternative therapeutic ways to treat CBM and other infections caused by *P. verrucosa*.

In the last years, metal-based drugs have been a subject of great interest due to their therapeutic values and pharmacological applications (Zhang and Lippard, 2003; Warra, 2011; Viganor et al., 2015). 1,10-Phenanthroline-5,6-dione (phendione) is a phenanthrene-based ligand and a derivative of the classical chelating agent 1,10-phenanthroline (Calderazzo et al., 2002; McCann et al., 2012a). Phendione has a structure similar to 1,10-phenanthroline with the addition of two carbonyl groups attached at positions 5 and 6. The bifunctional character of phendione made it an extremely versatile ligand, with special reactivity arising from its quinonoid and diiminic sites (Calderazzo et al., 2002; Calucci et al., 2006). The quinonoid functionality of phendione confers redox capability, whilst the juxtaposition is two *N* atoms make it ideally suited to chelating transition metal ions (Calderazzo et al., 2002; Calucci et al., 2006; McCann et al., 2012b). Phendione, both in its metal-free state and when coordinated to metal ions, is considered to have many interesting biological properties, such as anticancer and antimicrobial actions (McCann et al., 2004, 2012a; Deegan et al., 2006; Roy et al., 2008; Pivetta et al., 2014; Viganor et al., 2016). In recent years, researchers have synthesized several phendione-based compounds, including [Ag(phendione)_2_]ClO_4_ = [Ag(phendione)_2_]^+^ and [Cu(phendione)_3_](ClO_4_)_2_.4H_2_O = [Cu(phendione)_3_]^2+^ (McCann et al., 2004), in an attempt to generate new complexes with improved antimicrobial activity and reduced toxicity to different cell lineages, *Galleria mellonella* larvae and mice (McCann et al., 2012a). [Cu(phendione)_3_]^2+^ was found to be active against the multi-resistant, filamentous fungus, *Scedosporium apiospermum*, while [Ag(phendione)_2_]^+^ exhibited better activity against the yeast, *Candida albicans* (McCann et al., 2012a). Moreover, [Ag(phendione)_2_]^+^ caused extensive and non-specific DNA cleavage, disrupted cell division and caused severe morphological alterations in *C. albicans* yeast cells (Eshwika et al., 2004; McCann et al., 2012a). Metal-free phendione exerts its antimicrobial effect in several ways, such as disturbing the microorganism's crucial metal metabolism as well as interfering in its metal ion acquisition and its bioavailability for essential reactions (e.g., inhibiting the activity of metalloproteins), affecting the microbial cell homeostasis and culminating in the blockage of primordial biological events (e.g., nutrition, proliferation, differentiation, adhesion, invasion, dissemination and infection) (Santos et al., 2012). Protease inhibition is also a prime cellular target of this class of ligand and its associated metal complexes (Kellett et al., 2013). In this context, the aim of the present work was to investigate the effect of phendione and its Ag^+^ and Cu^2+^ complexes on *P. verrucosa* proliferation, ultrastructure, metallopeptidase activity, sterol content and morphogenesis.

## Materials and methods

### Chemicals

All reagents used in electrophoresis and buffers components were purchased from Bio-Rad (Hercules, CA, USA) and Merck (Darmstaldt, Germany). Human serum albumin (HSA), 1,10-phenanthroline, resazurin, AgClO_4_, Cu(ClO_4_)_2_·6H_2_O, dimethyl sulfoxide (DMSO), 3-(N-morpholino) propanesulfonic acid (MOPS), itraconazole (ITC), amphotericin B (AMB) ketoconazole (KTC), ergosterol, lanosterol, silica gel 60 plates, Czapek-Dox and Sabouraud-dextrose agar (SDA) components were obtained from Sigma-Aldrich Chemical Co (St Louis, MO, USA). Roswell Park Memorial Institute (RPMI) 1640 medium was purchased from Invitrogen (Camarillo, CA, USA). 1,10-Phenanthroline-5,6-dione (phendione), [Cu(phendione)_3_](ClO_4_)_2_·4H_2_O ([Cu(phendione)_3_]^2+^) and [Ag(phendione)_2_]ClO_4_ ([Ag(phendione)_2_]^+^) were prepared in accordance with published procedures (McCann et al., 2004).

### Microorganism and growth condition

*Phialophora verrucosa* (FMC.2214 strain) isolated from a human patient with CBM was grown on SDA medium. Fungal cells were cultivated for 7 days under constant agitation (130 rpm) at 26°C in 100 ml of Czapek-Dox, a chemically defined medium containing: 3 g sucrose; 0.3 g NaNO_3_; 0.05 g MgSO_4_.7H_2_O; 0.05 g KCl; 0.1 g KH_2_PO_4_; 0.001 g FeSO_4_.7H_2_O, pH 5.5. Conidia were obtained after gauze filtration followed by centrifugation (4.000 × g/10 min). Then, conidia were washed three times in 0.85% NaCl and cell density estimated by counting in a Neubauer chamber (Granato et al., 2015). All the experiments with *P. verrucosa* were conducted under Biosafety Level 2 (BSL-2) conditions.

### Effects of test compounds on *P. verrucosa* growth

Antifungal susceptibility testing was performed using the M38-A2 document for filamentous fungi as described by Clinical and Laboratory Standards Institute (CLSI, 2008) with some modifications (Granato et al., 2015). Briefly, the broth microdilution method was carried out using 96-well microtiter assay plates containing RPMI 1640 medium at pH 7.0 buffered with 0.16 M MOPS. All the test compounds were dissolved in DMSO and the maximum concentration of organic solvent was 2.0%. Serial dilution was made following CLSI guidelines in order to obtain final concentrations ranging from 0.01 to 20 mg/L of phendione and its derivatives, as well as the simple salts, AgClO_4_ and Cu(ClO_4_)_2_·6H_2_O. The minimum inhibitory concentration (MIC) for each test compound was determined after 5 days of incubation by visual inspection and resazurin staining assay (Liu et al., 2007). The lowest concentration capable in inhibiting 100% of fungal growth was recorded as the MIC. ITC (0.01 to 100 mg/L) was used as reference antifungal drug. In addition, the minimum fungicidal concentration (MFC) was established before microtiter plate (MIC assay) spectrophotometric reading, in which the contents of the plate wells were homogenized and an aliquot from each well was transferred onto SDA drug-free plates. The plates were incubated at 30°C for 10 days and MFC was determined as the lowest concentration without visual fungal growth. A fungicidal effect was defined as the MFC value equal or up to four times the MIC value, as proposed by Pfaller et al. (2004). The IC_50_ value, defined as the drug concentration (μM) able to cause a 50% reduction in fungal viability, was also calculated by using logarithmic regression after MIC determination, as detailed by Granato et al. (2015).

### Effects of test compounds on *P. verrucosa* ultrastructure

The fungal ultrastructure was evaluated using scanning electron microscopy (SEM). Briefly, conidia (5 × 10^7^ cells) were incubated for 20 h at 26°C in the absence (control) or in the presence of phendione, [Ag(phendione)_2_]^+^ or [Cu(phendione)_3_]^2+^ at concentrations corresponding to MIC and 2 × MIC. Subsequently, the conidia were washed and fixed with 4% paraformaldehyde and 2.5% glutaraldehyde in 0.15 M sodium cacodylate buffer (pH 7.2) at 26°C for 2 h. Cells were washed and then post-fixed for 1 h at 26°C with 1% OsO_4_ in the same buffer. Next, samples were dehydrated using a graded series of ethanol (50–100%), and dried by the critical point method. Finally, the samples were mounted on stubs, coated with gold and observed using a Jeol JSM 6490LV scanning electron microscope (Abi-chacra et al., 2013).

### Effects of combinations of [Cu(phendione)_3_]^2+^ with classical antifungals on *P. verrucosa* growth

All the test compounds were dissolved in DMSO. *P. verrucosa* conidia (1 × 10^3^/ml) were exposed to individual clinically used antifungal drugs [AMB (3.12 mg/L), KTC (1.56 mg/L) and ITC (0.78 mg/L) at concentration values corresponding to ½ × MIC of each drug] and also to [Cu(phendione)_3_]^2+^ (½ × MIC). In addition, combinations of separate samples of AMB, KTC and ITC with [Cu(phendione)_3_]^2+^ at these concentrations were also screened for 1 h at 26°C. After exposure, 0.1 ml of each system was plated onto solid Czapek-Dox medium without drugs and incubated for 5 days at 26°C. Fungal growth was estimated by counting colony-forming units (CFU) (Palmeira et al., 2008) and the results were compared to the untreated control.

### Effects of test compounds on the metallopeptidase activity of *P. verrucosa*

*P. verrucosa* conidial cells (5 × 10^9^) were resuspended in 0.2 ml of sterile phosphate-buffered saline (PBS, pH 7.2) supplemented with 2% glucose and incubated with constant agitation (130 rpm). After 2 h, conidia were removed by centrifugation (4000 × g/10 min) and the cell-free PBS-glucose supernatant was subjected to a peptidase activity assay as described by Granato et al. (2015). Briefly, 15 μl of cell-free PBS-glucose supernatant (10 μg of protein) and 1.5 μl HSA (1 mg/ml) were incubated for 20 h at 37°C in the absence (control) or in the presence of the classical metallopeptidase inhibitor, 1,10-phenanthroline (10 mM), and 8 × MIC of the test compounds, phendione, [Ag(phendione)_2_]^+^ and [Cu(phendione)_3_]^2+^. The reaction mixtures were then added to 15 μl sample buffer (125 mM Tris, pH 6.8, 4% SDS, 20% glycerol, 0.002% bromophenol blue and 10% β-mercaptoethanol), boiled at 100°C for 5 min, and subjected to sodium dodecyl sulfate-polyacrylamide gel electrophoresis (SDS-PAGE). Electrophoresis was carried out at 4°C, 120 V for 1.5 h. The degradation protein profiles were detected by silver staining (Granato et al., 2015). Densitometric quantification was performed using the free ImageJ software.

### Effects of test compounds on sterol content

Conidia (1 × 10^7^/ml) were incubated in Czapek-Dox medium in the absence (control) or in the presence of sub-inhibitory concentrations (½ × MIC and ¼ × MIC) of phendione, [Ag(phendione)_2_]^+^ or [Cu(phendione)_3_]^2+^. After 48 h, conidia were washed in PBS and total lipids were extracted with chloroform:methanol (2:1, 1:1 and 1:2). The combined extracts were mixed, dried and Folch partition was then performed (Folch et al., 1957). The lower phase containing the neutral lipids was recovered, evaporated and subjected to high performance thin layer chromatography (HPTLC). Chromatography was carried out on silica gel 60 plates, which were developed with a solvent system containing hexane:ether:acetic acid (60:30:1.5). The spots (violet-red color) were visualized after spraying the plate with a reagent (comprising 50 mg iron chloride, 5 ml sulfuric acid, 5 ml acetic acid and 90 ml distilled water) and subsequent heating (Larsen et al., 2004). The sterol standards, ergosterol (4 μg) and lanosterol (1 μg) were used. Sterol quantitative determination was performed using ImageJ software.

### Effects of test compounds on *P. verrucosa* morphogenesis

Conidia (1 × 10^6^/ml) were incubated at 26°C in the absence (control) or in the presence of sub-inhibitory concentrations (½ × MIC and ¼ × MIC) of phendione, [Ag(phendione)_2_]^+^ or [Cu(phendione)_3_]^2+^ in RPMI medium, pH 7.0 (without agitation), in order to induce the filamentation in *P. verrucosa*. The fungal cells were then observed using a Carl Zeiss MicroImaging GmbH optical microscope and images obtained every 24 h (Granato et al., 2015). Conidial viability after the treatment with the test compounds was performed using the resazurin assay (Liu et al., 2007).

### Statistical analysis

All experiments were performed in triplicate in three independent experimental sets. The graphics and data were constructed and analyzed statistically by means of Student's *t*-test using GraphPad Prism 5.01 software. *P* values of 0.05 or less were assumed as significant.

## Results and discussion

### Anti-*P. verrucosa* action of phendione and its metal complexes

**Figure 1:**
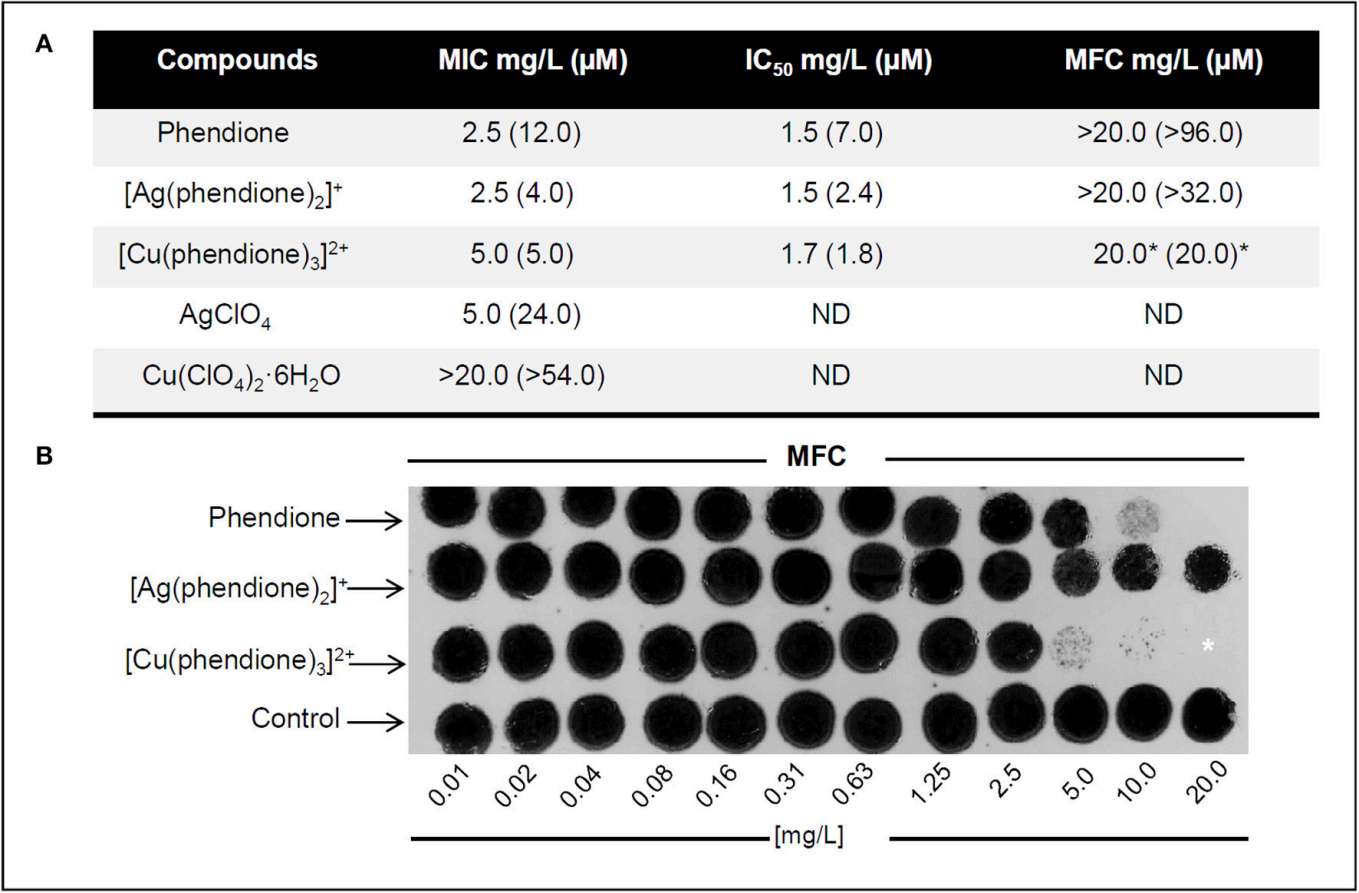
**Effect of test compounds on *P. verrucosa* viability. (A)** Antifungal effects of phendione, [Ag(phendione)_2_]^+^, [Cu(phendione)_3_]^2+^ and the simple salts, AgClO_4_ and Cu(ClO_4_)_2_·6H_2_O. **(B)** MFC representative images of phendione and its Ag^+^ and Cu^2+^ complexes on *P. verrucosa* growth. **(A,B)** Fungicidal effect was considered when the MFC value was either equal to or up to 4 × MIC value for a compound (Pfaller et al., 2004), which was indicated by asterisk (^*^). ND, Not determined.

The *in vitro* antifungal activities of phendione and its Ag^+^ and Cu^2+^ complexes were evaluated against *P. verrucosa*. All of the test compounds inhibited conidial cell growth with the following activity order based on the IC_50_ values (μM): [Cu(phendione)_3_]^2+^ > [Ag(phendione)_2_]^+^ > phendione (Figure 1). Only [Cu(phendione)_3_]^2+^ showed a fungicidal effect. These data corroborate previously published results, which revealed that phendione-based metal complexes had higher antimicrobial activity than metal-free phendione toward different classes of microorganisms (McCann et al., 2012a; Viganor et al., 2016).

In order to confirm that the cytotoxic effect observed was due to the complexes, rather than the free Ag^+^ and Cu^2+^ ions, the antifungal activities of the simple perchlorate salts, Cu(ClO_4_)_2_·6H_2_O and AgClO_4_, were also assessed (Figure 1). While AgClO_4_ showed moderate anti-*P. verrucosa* activity (MIC 24.0 μM), Cu(ClO_4_)_2_·6H_2_O did not affect fungal growth, even at the maximum test concentration (MIC > 54.0 μM). The results showed that Ag^+^ coordinated to phendione ([Ag(phendione)_2_]^+^) was about 6 times more effective (MIC 4.0 μM) than simple AgClO_4_ (MIC 24.0 μM). Although, Cu(ClO_4_)_2_·6H_2_O was essentially inactive (MFC > 54.0 μM), Cu^2+^ coordinated to phendione ([Cu(phendione)_3_]^2+^) was able to inhibit 100% *P. verrucosa* proliferation at 5.0 μM. It is believed that the phendione metal complexes have a higher lipophilicity than the simple metal salts (Viganor et al., 2016). This property is due to the total electron density reduction on the free ligand upon complexation to the metal ion, and also to the sharing of the positive charge of the metal cation with N-donor atoms of the phendione ligand, which promotes an electron delocalization all over the chelate ring (Raman et al., 2014; Viganor et al., 2016). Aqueous DMSO, which was used as the solvent for all of the test compounds, was inactive against *P. verrucosa* conidial proliferation (data not shown). Altogether, the present *P. verrucosa* growth inhibition results suggest that the activity of the metal complexes as a whole is superior to that of either the free metal ion or metal-free phendione ligand. Ag^+^ (d^10^ outer electron configuration) complexes have zero ligand field stabilization energy (LFSE) whilst octahedral Cu^2+^ (d^9^) has only a small amount of LFSE. As such, Ag^+^ and Cu^2+^ complexes are expected to be labile and the original chelating phendione ligands are expected to be rapidly exchanged for biological ligands present within the fungal cells and also in the growth medium (amino acids, proteins, ammonia, chloride etc.). It is thus likely that cell growth inhibition arises due to the destructive interference of cellular processes by the metal ion coupled with inactivation of other cell events by the phendione ligand. It also appears that it is the phendione ligand component of the administered metal complex that plays the dominant role in the demise of the conidia.

Our previous results described that *P. verrucosa* was also sensitive to 1,10-phenanthroline treatment, showing MIC equal to 4.4 μM (0.8 μg/ml) (Granato et al., 2015). Thus, the antifungal activity of 1,10-phenanthroline was superior to metal-free phendione, but similar to its Ag^+^ and Cu^2+^ complexes. However, neither 1,10-phenanthroline nor phendione presented fungicidal effect against *P. verrucosa* (Granato et al., 2015).

### Ultrastructural alterations induced by phendione and its metal derivatives

**Figure 2:**
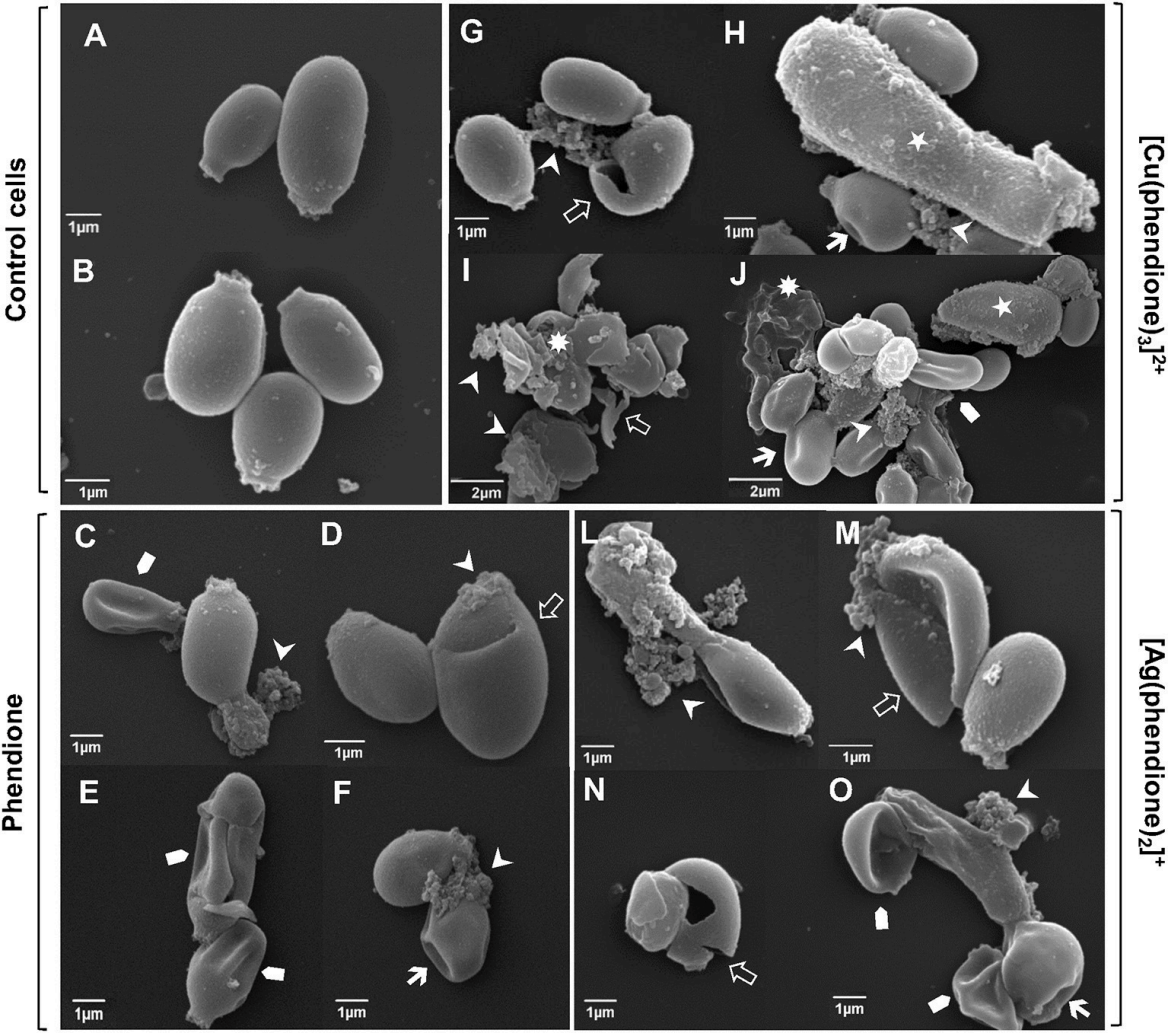
**Effect of test compounds on *P. verrucosa* ultrastructure**. Representative SEM images showing untreated cells (control systems, **A,B**) and conidial cells treated with phendione **(C–F)**, [Cu(phendione)_3_]^2+^ **(G–J)** and [Ag(phendione)_2_]^+^ **(L–O)** at both MIC **(C,D,G,H,L,M)** and 2 × MIC **(E,F,I,J,N,O)**. In contrast to untreated conidial cells, treatment with the test compounds induced several ultrastructural alterations, including cell size increase (⋆), surface invaginations (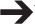), cell disruption (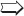), surface detachment (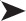), cellular debris (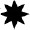) and cell shrinkage (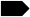).

The effect of phendione, [Ag(phendione)_2_]^+^ and [Cu(phendione)_3_]^2+^ on *P. verrucosa* ultrastructure was probed using SEM. In contrast to control cells, which had typical spherical-to-oval morphology (Figures 2A,B), cells exposed to the test compounds exhibited several surface alterations, such as detachment of cell wall components, invaginations, cellular disruptions and shrinkages (Figures 2C–O), which are indicative of cell death. [Cu(phendione)_3_]^2+^ was the most aggressive in promoting changes on both conidial architecture and morphology, for example, inducing cell size increases when treated at the MIC concentration (Figure 2H) and cellular debris following incubation with 2 × MIC (Figures 2I,J).

Our previous study showed that 1,10-phenanthroline (25 μM) also caused irreversible ultrastructure alterations on *P. verrucosa* conidia, including cell wall detachment, intense intracellular vacuolization and reduction in the cytoplasm electron density (Granato et al., 2015). Similarly, phendione and [Ag(phendione)_2_]+, both at 10 μg/ml, promoted changes in the internal structure of *C. albicans*, as observed by transmission electron microscopy (McCann et al., 2004). In that work, phendione promoted an increase in size as well as a reduction in the budding process of *C. albicans* yeast cells. Furthermore, *C. albicans* exhibited a diffuse cell wall, rupture of internal organelles and nucleus enlargement following treatment with phendione. *C. albicans* yeast cells treated with [Ag(phendione)_2_]^+^ had a distended cell wall, rupture of membranous organelles and, in some cases, a fragmented nucleus. Phendione and its Ag^+^ complex were both able to cause a withdrawal of the cytoplasmic membrane from within the cell wall in *C. albicans* (McCann et al., 2004).

### Combination of [Cu(phendione)_3_]^2+^ with AMB induces a decrease in the viability of *P. verrucosa*

**Figure 3:**
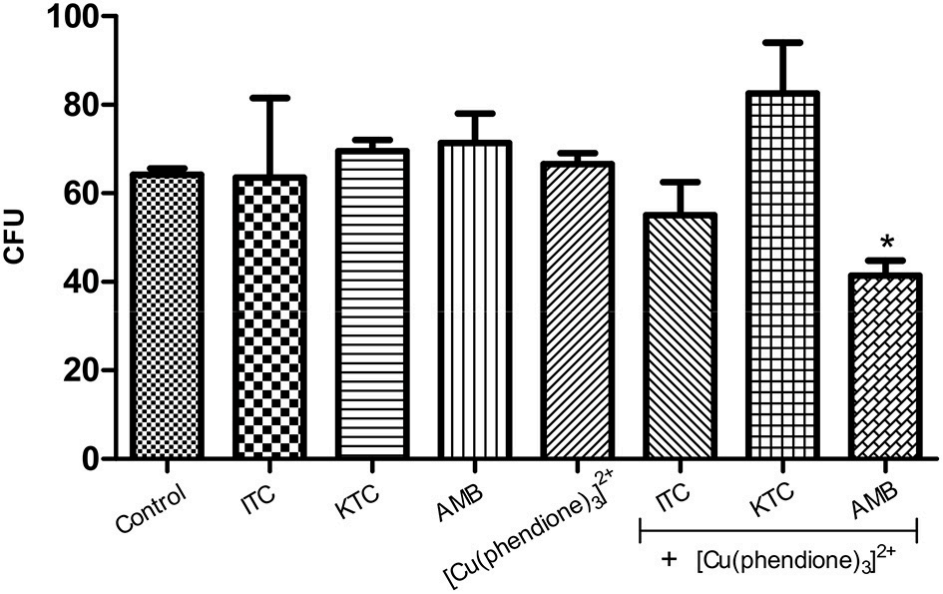
**Effect of a combination of [Cu(phendione)_3_]^2+^ with classical antifungal agents on *P. verrucosa* development**. Conidia were untreated (control) or treated with a sub-inhibitory concentration (½ × MIC) of [Cu(phendione)_3_]^2+^ and classical antifungal drugs (AMB, ITC and KTC), alone or in combination. After 1 h, conidia were inoculated in a fresh solid Czapek-Dox medium to measure the CFU. Conidia treated only with DMSO (compound diluent) did not alter the cell growth. The values represent the mean ± standard deviation of three independent experiments performed in triplicate. Symbol (^*^) denotes the system that had a growth rate significantly different from the control (*P* < 0.05, Student's *t* test).

In this set of experiments, the most potent test compound, [Cu(phendione)_3_]^2+^, was chosen to be combined with a selection of classical antifungal agents in an attempt to check their ability to control *P. verrucosa* growth. In this context, the combination of [Cu(phendione)_3_]^2+^ with AMB, both of which were deployed at (½ × MIC) concentrations, was able to significantly inhibit the fungal proliferation by around 40% (Figure 3). However, at the concentrations used, [Cu(phendione)_3_]^2+^ did not positively compliment the activities of either KTC or ITC, since *P. verrucosa* growth was not affected by these combinations (Figure 3).

Eshwika and coworkers [22] showed that pre-growth of *C. albicans* with sub-inhibitory concentrations (¼ × MIC_90_) of [Ag(phendione)_2_]^+^ affected the subsequent susceptibility of this yeast to miconazole and AMB, reducing the concentrations of these clinical antifungal agents required to achieve MIC_90_. Benefits of combination therapy are well-known and include broad spectrum efficacy, greater potency compared to monotherapy, improvements in both safety and tolerability as well as a reduction in the emergence of resistance (Cuenca-Estrella, 2004; Spitzer et al., 2016). Metal-based drugs can have modes of action distinct from classical antifungal agents, allowing their use in cases where there is resistance to conventional therapies. Furthermore, different mode(s) of action can be used by employing such metal-containing drugs in combination with existing antifungals in order to target two (or more) sites in the fungal cells. Thus, there is the potential of achieving the same therapeutic effect by reducing the concentration of clinical drugs used (Eshwika et al., 2004).

### Phendione-based compounds modulate the metallopeptidase activity of *P. verrucosa*

**Figure 4:**
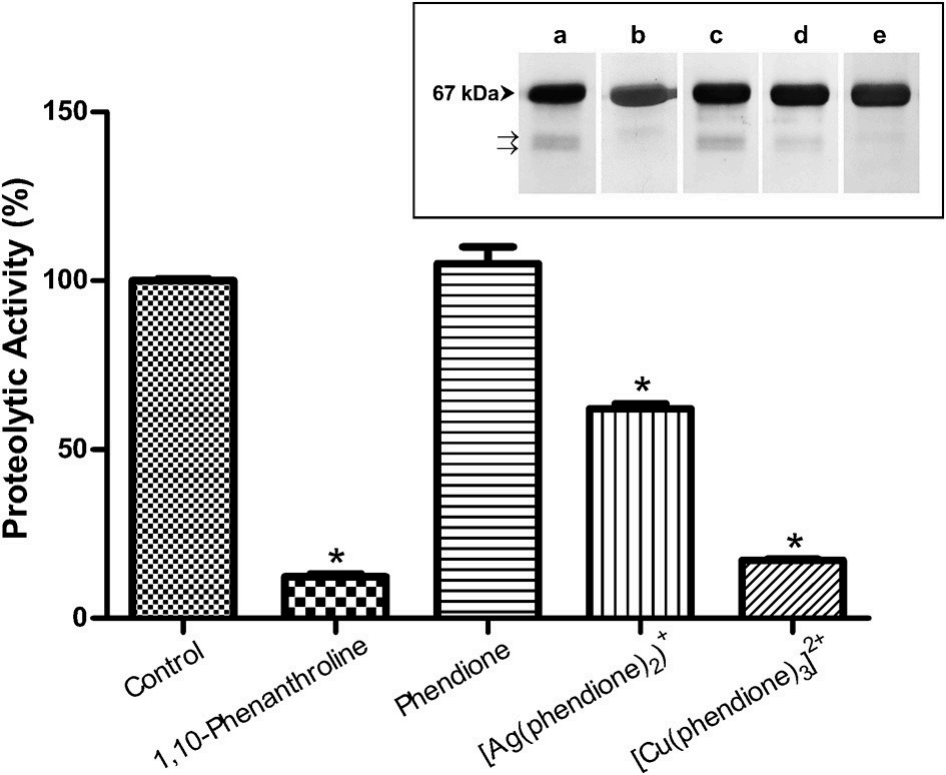
**Effects of phendione and its derivatives on *P. verrucosa* metallopeptidase activity**. PBS-glucose supernatant was incubated at 37°C in 20 mM sodium acetate buffer, pH 3.0, and HSA in the absence (control) or in the presence of phendione and its metal complexes at concentrations corresponding to 8 × MIC value. After 20 h, the supernatant was subjected to the proteolytic activity assay, as detailed in Material and Methods. The graphic shows densitometric quantification of the bands observed in the SDS-PAGE (*inset*), which was performed using the software ImageJ. The HSA fragment of the control system was taken as 100%. Symbols (^*^) indicate the experimental systems considered statistically significant from the control (*P* < 0.05, Student's *t*-test). *Inset*: representative images of SDS-PAGE, in which *P. verrucosa* supernatant was treated with 1,10-phenantroline at 10 mM **(b)** and 8 × MIC of all other compounds, such as: phendione **(c)**, [Ag(phendione)_2_]^+^ **(d)** and [Cu(phendione)_3_]^2+^ **(e)**. Control system contained only HSA and PBS-glucose supernatant **(a)**. The number on the left represents the HSA molecular mass, expressed in kDa. The arrows (→) show fragmentation of the proteinaceous substrate after proteolysis.

[Cu(phendione)_3_]^2+^ and [Ag(phendione)_2_]^+^ were capable of inhibiting *P. verrucosa* extracellular metallopeptidase activity by around 85 and 40%, respectively (Figure 4). However, the metal-free ligand, phendione, did not affect this enzymatic activity (Figure 4). The presence of metallopeptidase in *P. verrucosa* PBS-glucose supernatant was confirmed by its inhibition by 10 mM of 1,10-phenanthroline (Figure 4).

Metal-chelating-type compounds may affect typical functions of several eukaryotic proteins, such as various metallo-type enzymes, including metallopeptidases (McCann et al., 2012b; Santos et al., 2012; Granato et al., 2015). In fact, the inhibition of metallopeptidases by 1,10-phenanthroline occurs mainly due to its ability of promoting Zn^2+^ chelation, which is required for catalytic activity and which leaves an inactive apoenzyme (Santos et al., 2012). Our research group showed previously that Zn^2+^ ions were able to stimulate the enzymatic activity of *P. verrucosa* extracellular peptidase in a typical dose-dependent manner, suggesting the presence of Zn^2+^-metallopeptidase in this fungus (Granato et al., 2015). It is well-known that metallopeptidase inhibition can prevent fungal cells to obtain necessary peptides and amino acids for nutrition, leading to reduction or complete inhibition of cell growth (Santos et al., 2012).

### Phendione and its metal complexes inhibit sterol content of *P. verrucosa* conidia

**Figure 5:**
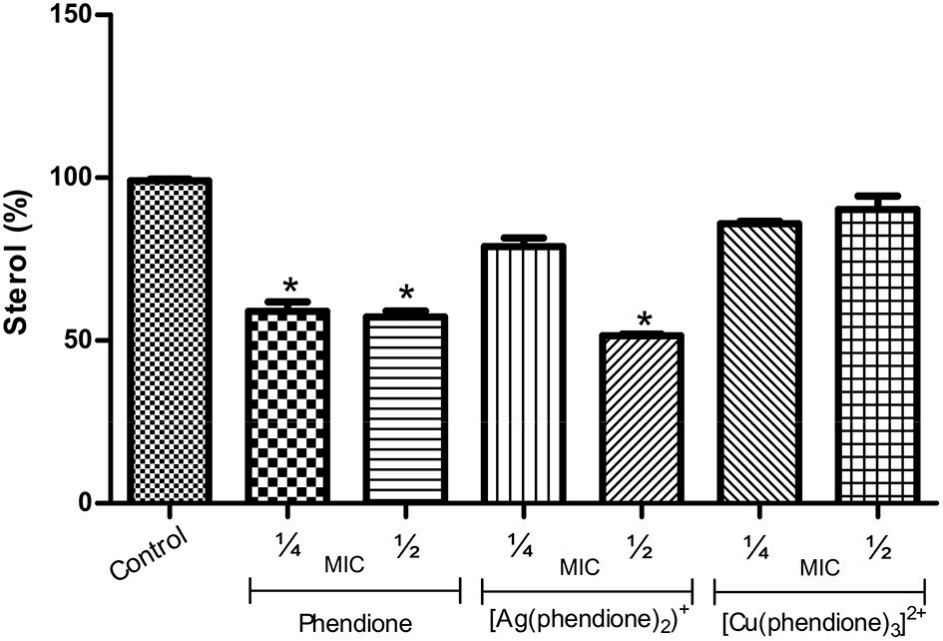
**Effects of phendione and its metal complexes on *P. verrucosa* sterol content**. Conidia (1 × 10^7^ cells/ml) were either untreated (control) or treated for 48 h in the presence of sub-inhibitory concentrations (¼ × MIC and ½ × MIC) of each test compound. Afterwards, lipids were extracted, and neutral lipids were applied onto HPTLC plates. Graphics represent densitometric quantifications of results obtained by HPTLC using the software ImageJ. The sterol content of the control system was taken as 100%. Asterisk (^*^) denotes significant differences (*P* < 0.05, Student's *t* test) between treated systems and untreated cells (control).

The treatment of conidial cells with phendione (¼ × MIC and ½ × MIC) and also with [Ag(phendione)_2_]^+^ (½ × MIC) promoted a reduction in ergosterol content by approximately 50% in relation to the control (untreated) cells (Figure 5). On the contrary, [Cu(phendione)_3_]^2+^ did not affect the sterol content of *P. verrucosa* (Figure 5).

Previous studies showed that sterol synthesis in *C. albicans* was also disturbed by phendione and its metal complexes (Eshwika et al., 2004). Interestingly, and in contrast to our results, [Cu(phendione)_3_]^2+^ diminished the ergosterol content in *C. albicans* yeast cells, while [Ag(phendione)_2_]^+^ enhanced the amount of this lipid level (Eshwika et al., 2004). It is well-known that sterol deficiency disturbs crucial cell membrane properties, leading to an increased fluidity and permeability, which may cause severe structural aberrations that contributes to cell death (Kathiravan et al., 2012).

### Phendione and its metal complexes affect fungal morphological transition

**Figure 6:**
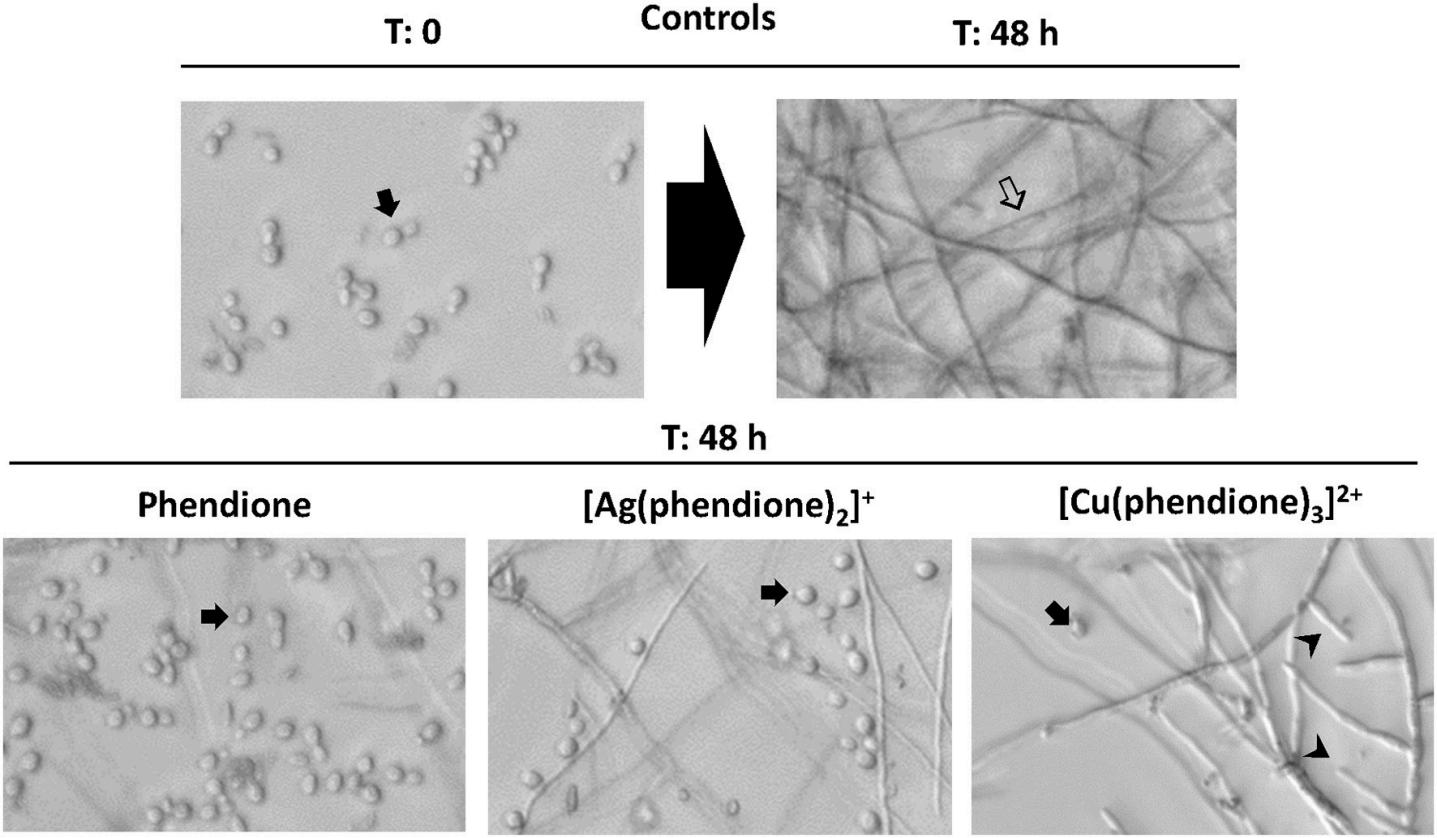
**Effects of phendione and its metal complexes on *P. verrucosa* filamentation**. Conidia (1 × 10^6^ cells/ml) were grown in RPMI medium in either the absence (control) or the presence of sub-inhibitory concentration (¼ × MIC) of phendione, [Ag(phendione)_2_]^+^ and [Cu(phendione)_3_]^2+^. Conidia were observed before (*T* = 0: zero time) and after 48 h of incubation (*T* = 48 h) by optical microscopy, when the fungal filamentous form (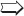) was produced. The inhibition of *P. verrucosa* mycelial development by compounds was shown by conidia (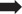) observation and hyphal branch (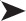) growth reduction.

The present studies demonstrated that all the test compounds were able to effectively block to a large extent the morphological transition (conidia-into-mycelial transformation) when compared to untreated fungal cells (Figure 6). It is important to notice that hyphal formation was mostly inhibited by phendione, followed by [Ag(phendione)_2_]^+^ and then by [Cu(phendione)_3_]^2+^ (Figure 6). In fact, several conidia (instead of hyphae form) were observed after the treatment with phendione and [Ag(phendione)_2_]^+^, both used at a sub-inhibitory concentration (¼ × MIC). It was also possible to observe some conidial cells in the [Cu(phendione)_3_]^2+^ system (¼ × MIC); however, the highest effect of this compound was its considerable ability to reduce hyphal branch growth (Figure 6).

Morphological transition is one of the strategies used by several fungi to survive in environment and in vertebrate hosts (Wang and Lin, 2012). In this context, several studies have shown that cell differentiation is an essential step in the establishment and success of fungal infection (Jacobsen et al., 2012; Wang and Lin, 2012). We have previously demonstrated that chelating compounds, such as 1,10-phenanthroline, were able to inhibit the conidia-into-hyphae transformation in *P. verrucosa* (Granato et al., 2015). Other studies also reported the same capacity of 1,10-phenanthroline to affect cell differentiation in *C. albicans, Pseudallescheria boydii*, and *Fonsecaea pedrosoi* (Santos et al., 2012). The mechanisms involved in the morphological transition of *P. verrucosa* have not been established. It is well-known that several factors are associated with fungal morphogenesis, including nutritional elements, temperature and aerobic conditions (Wang and Lin, 2012). Mendoza et al. (1993) showed that CBM fungi, including *P. verrucosa* produced large numbers of sclerotic bodies after inoculation into a defined pH 2.5 medium containing the metal ion Ca^2+^ at 0.1 mM concentration. While higher concentrations of Ca^2+^ (1 mM) reversed this pattern and promoted maintenance of *P. verrucosa* hyphal growth. Addition of the Ca^2+^ chelator, ethylene glycol tetraacetic acid (EGTA, 8 mM) to the culture medium induced *P. verrucosa* sclerotic bodies, demonstrating the importance of metal ion Ca^2+^ in this fungal essential process (Mendoza et al., 1993). In this context, these results revealed that metal ion chelating agents can modulate morphological transitions in CBM fungi.

## Conclusions

Metal-free phendione and its Ag^+^ and Cu^2+^ complexes are able to arrest the growth of *P. verrucosa*, especially [Cu(phendione)_3_]^2+^, which also presented a fungicidal action. In addition, these compounds blocked some vital fungal events, such as filamentation, as well as reducing both sterol production and the activity of metallo-type peptidase. Previous studies reported that metal-based drugs showed tolerable toxicity *in vivo*, confirming that they represent a novel group of antifungal agents. Moreover, these compounds could be used alone or in combination with classical antifungal agents, since *P. verrucosa* had its growth inhibited after the combined treatment with [Cu(phendione)_3_]^2+^ and AMB, as suggested in this study. In conclusion, our data point out that metallo-drugs have potential applications for the control and treatment of *P. verrucosa* infections.

## Author contributions

MG, MM, MD, AS, and LK conceived and designed the study. MG, DG, and SS performed the experiments. All authors analyzed the data. MM, MD, AS, and LK contributed reagents, materials and/or analysis tools. MG, MM, MD, AS, and LK wrote and revised the paper. All authors contributed to the research and approved the final version of the manuscript. All authors agree to be accountable for all aspects of the work.

## Funding

This study was supported by grants from the Brazilian agencies: Conselho Nacional de Desenvolvimento Científico e Tecnológico (CNPq), Fundação de Amparo à Pesquisa no Estado do Rio de Janeiro (FAPERJ), Coordenação de Aperfeiçoamento de Pessoal de Nível Superior (CAPES) and Fundação Oswaldo Cruz (FIOCRUZ).

### Conflict of interest statement

The authors declare that the research was conducted in the absence of any commercial or financial relationships that could be construed as a potential conflict of interest.
